# Lipid levels in midlife and risk of atrial fibrillation over 3 decades—Experience from the Swedish AMORIS cohort: A cohort study

**DOI:** 10.1371/journal.pmed.1004044

**Published:** 2022-08-11

**Authors:** Mozhu Ding, Alexandra Wennberg, Bruna Gigante, Göran Walldius, Niklas Hammar, Karin Modig

**Affiliations:** 1 Unit of Epidemiology, Institute of Environmental Medicine, Karolinska Institutet, Stockholm, Sweden; 2 Division of Cardiovascular Medicine, Department of Medicine, Karolinska Institutet and Division of Cardiology, Danderyd University Hospital, Stockholm, Sweden; Harvard Medical School, UNITED STATES

## Abstract

**Background:**

The role of cholesterol levels in the development of atrial fibrillation (AF) is still controversial. In addition, whether and to what extent apolipoproteins are associated with the risk of AF is rarely studied. In this study, we aimed to investigate the association between blood lipid levels in midlife and subsequent risk of new-onset AF.

**Methods and findings:**

This population-based study included 65,136 individuals aged 45 to 60 years without overt cardiovascular diseases (CVDs) from the Swedish Apolipoprotein-Related Mortality Risk (AMORIS) cohort. Lipids were measured in 1985 to 1996, and individuals were followed until December 31, 2019 for incident AF (i.e., study outcome). Hazard ratios (HRs) with 95% confidence intervals (CIs) were estimated using Cox regression, adjusting for age, sex, and socioeconomic status. Over a mean follow-up of 24.2 years (standard deviation 7.5, range 0.2 to 35.9), 13,871 (21.3%) incident AF cases occurred. Higher levels of total cholesterol (TC) and low-density lipoprotein cholesterol (LDL-C) were statistically significantly associated with a lower risk of AF during the first 5 years of follow-up (HR = 0.61, 95% CI: 0.41 to 0.99, *p* = 0.013; HR = 0.64, 95% CI: 0.45 to 0.92, *p* = 0.016), but not thereafter (HR ranging from 0.94 [95% CI: 0.89 to 1.00, *p* = 0.038] to 0.96 [95% CI: 0.77 to 1.19, *p* > 0.05]). Lower levels of high-density lipoprotein cholesterol (HDL-C) and apolipoprotein A-I (ApoA-I) and higher triglycerides (TG)/HDL-C ratio were statistically significantly associated with a higher risk of AF during the entire follow-up (HR ranging from 1.13 [95% CI: 1.07 to 1.19, *p* < 0.001] to 1.53 [95% CI: 1.12 to 2.00, *p* = 0.007]). Apolipoprotein B (ApoB)/ApoA-I ratio was not associated with AF risk. The observed associations were similar among those who developed incident heart failure (HF)/coronary heart disease (CHD) and those who did not. The main limitations of this study include lack of adjustments for lifestyle factors and high blood pressure leading to potential residual confounding.

**Conclusions:**

High TC and LDL-C in midlife was associated with a lower risk of AF, but this association was present only within 5 years from lipid measurement and not thereafter. On the contrary, low HDL-C and ApoA-I and high TG/HDL-C ratio were associated with an increased risk of AF over almost 35 years of follow-up. ApoB/ApoA-I ratio was not associated with AF risk.

## Abstract 2

Mozhu Ding and colleagues investigate investigate the association between blood lipid levels in midlife and subsequent risk of new-onset atrial fibrillation in Sweden.

## Author summary

**Why was this study done?:**

- High cholesterol level is an established risk factor for cardiovascular diseases (CVDs), such as coronary heart disease (CHD).
- However, the role of cholesterol levels in the development of atrial fibrillation (AF), a very common cardiac arrhythmia in older adults, is debated.
- Compared to cholesterols, apolipoprotein B (ApoB), apolipoprotein A-I (ApoA-I), and their ratios have been shown to better predict CVD, but whether and how they are associated with AF is unclear.

**What did the researchers do and find?:**

- In this large Swedish cohort with blood-based biomarker information, 65,136 individuals free of overt CVD and aged 45 to 60 years at lipid measurement were followed for up to 35 years for new-onset AF.
- High levels of total cholesterol (TC) and low-density lipoprotein cholesterol (LDL-C) were associated with a lower risk of AF (hazard ratio, HR = 0.61, 95% confidence interval (CI): 0.41 to 0.99, *p* = 0.013; HR = 0.64, 95% CI: 0.45 to 0.92, *p* = 0.016), but this association was present only during the first 5 years since lipid measurement.
- On the contrary, low levels of high-density lipoprotein cholesterol (HDL-C) and ApoA-I and a high triglyceride (TG)/HDL-C ratio were associated with an increased risk of AF throughout the follow-up time (HR ranging from 1.13 [95% CI: 1.07 to 1.19, *p* < 0.001] to 1.53 [95% CI: 1.12 to 2.00, *p* = 0.007]). High ApoB/ApoA-I ratio was not associated with the risk of subsequent AF.

**What do these findings mean?:**

- Dyslipidemia expressed by a high TC or LDL-C, or a poor atherogenic to anti-atherogenic balance, does not seem to be associated with an increased risk of developing AF.
- Instead, the findings suggest that metabolic syndrome and inflammation, as indicated by low HDL-C and ApoA-I and a high TG/HDL-C ratio, may play a role in the onset of AF.

## Introduction

Atrial fibrillation (AF) is the most common cardiac arrhythmia in older adults, leading to individual suffering and large healthcare expenditures. Several factors including old age, sex, diabetes, obesity, and hypertension are associated with an increased risk of AF [1,2]. Many of these factors are also associated with coronary heart disease (CHD) and heart failure (HF), which often precedes the onset of AF. Lipid biomarkers are established strong risk factors for CHD, yet whether and how lipid levels play a role in the development of AF is not entirely clear.

Only a few prospective cohort studies have examined the association between cholesterol levels and risk of AF, where some unexpectedly found a lower AF incidence among people with higher levels of total cholesterol (TC) or low-density lipoprotein cholesterol (LDL-C) [3–5]. For triglycerides (TGs), most studies found no association with AF risk [3,6,7]. Synthesis of previous evidence has been hampered by differences in study population, characteristics, different follow-up time, lack of subgroup analysis (e.g., co-occurring cardiovascular diseases (CVDs)), and varying methods for laboratory analyses on biomarkers. In addition, there is even less information and greater uncertainty concerning the association between apolipoproteins and risk of AF. Apolipoprotein B (ApoB) and apolipoprotein A-I (ApoA-I) reflect the total number of atherogenic and anti-atherogenic particles and have been shown to better predict CVD than conventional lipid indicators [8,9]. Studying ApoB, ApoA-I, and their ratio, could therefore provide more insights into the role of lipid metabolism in the onset of AF than studying cholesterols alone.

In the current study, we have access to an exceptionally large biomarker dataset, the Swedish AMORIS (Apolipoprotein MOrtality RISk) cohort, to explore the association between blood lipid levels in midlife (45 to 60 years) and risk of new-onset AF later in life. The large sample size and a follow-up for up to 35 years enable the possibility to perform subgroup analyses to examine (1) whether the associations differ by co-occurring HF/CHD; and (2) whether the associations differ by duration of follow-up.

## Methods

The AMORIS cohort is a population-based study initiated to assess the role of common blood-based biomarkers in the development of chronic diseases [10,11]. A total of 812,073 Swedish men and women were enrolled in the cohort between 1985 and 1996 (baseline period), whose blood samples were drawn for laboratory analysis on a varying number of biomarkers. The majority of the participants were residing in the greater Stockholm area at the time of blood sampling. All blood samples were obtained through either a general health check-up or a referral from occupational or other outpatient care during the enrollment period. All laboratory analyses were performed on fresh blood samples by the Central Automation Laboratory (CALAB), Stockholm, Sweden, using a consistently implemented and well-documented methodology [11].

Using the unique Swedish personal identification numbers that were assigned to all Swedish residents at the time of birth or immigration, the AMORIS cohort is linked to a number of national registers including the National Patient Register, Cause of Death Register, Total Population Register, National Census, and Prescribed Drug Register, to provide information on socioeconomic status (SES), hospital and specialized outpatient care diagnoses, date of death and cause of death, emigration status, and prescribed medications. The National Census was mandatory for adult Swedish citizens every fifth year from 1960 up until 1990 and includes questions on employment status and occupation. Based on this information, an index of SES was defined according to the socioeconomic classification system elaborated by Statistics Sweden, where all individuals were classified into 6 categories: skilled/unskilled laborers, lower-level employees, middle-level employees, higher-level employees, self-employed, and others. All participants were followed from the date of their first laboratory test until the date of AF diagnosis, date of death, emigration, or end of follow-up (December 31, 2019), whichever came first.

**Fig 1:**
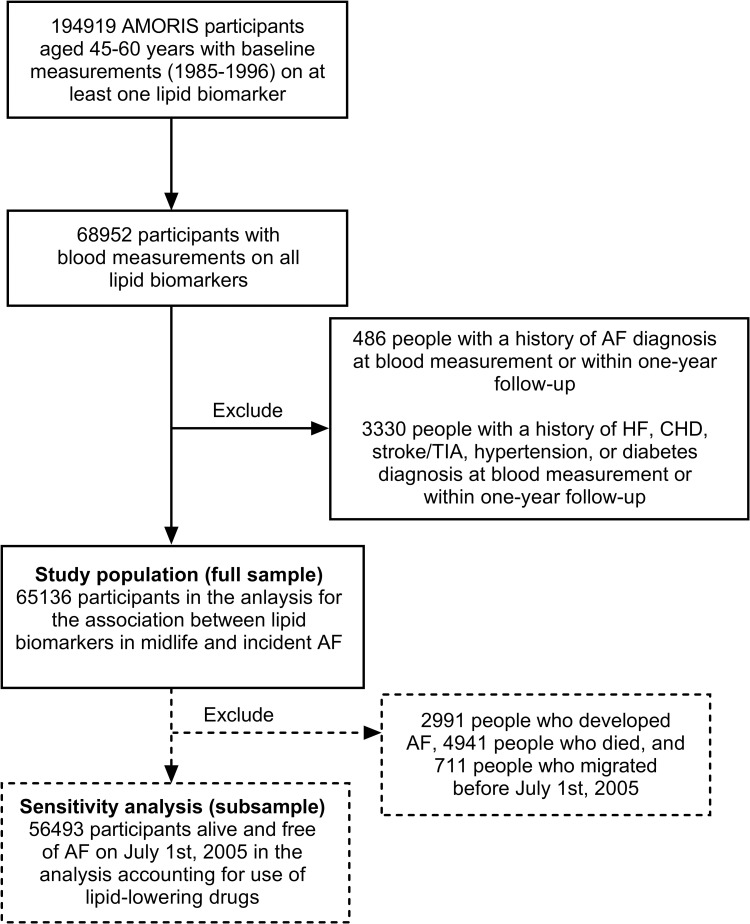
Flow chart of the study population in the AMORIS cohort. AF, atrial fibrillation; AMORIS, Apolipoprotein-Related Mortality Risk; CHD, coronary heart disease; HF, heart failure; TIA, transient ischemic attack.

Fig 1 shows the flowchart of the final study population. A total of 194,919 participants aged 45 to 60 years (born between 1924 to 1945) at the time of blood measurements were eligible for inclusion in the current study. For the analysis on biomarkers to be comparable with each other, participants in the study sample needed to have information on the blood measurement date for all lipid biomarkers used in this study. Therefore, 112,833 persons who were missing blood measurement on at least 1 of the studied lipid biomarkers were excluded. To include only incident cases of AF and to account for reverse causality, 486 people who had previous diagnosis of AF at baseline or had new AF diagnosis within the first year of follow-up were further excluded. Another 3,330 people with a history of HF, CHD, stroke, transient ischemic attack (TIA), hypertension, or diabetes at baseline or had new such diagnoses within the first year of follow-up were additionally excluded. The full study sample consists of 65,136 individuals free of overt CVD aged 45 to 60 years at blood measurement to study the association between lipid levels and incident AF. In addition, to assess the role of lipid-lowering medications for which information is only available from July 2005 onward, a subsample of 56,493 people alive and free of AF on July 1, 2005 was used.

This study complies with the Declaration of Helsinki and has been approved by the Regional Ethical committee at Karolinska Institutet, Stockholm, Sweden (reference number 2018/2401-31). The ethical board waived the need for informed consent because of the large number of participants in the cohort and because half of them had already died at the time of linkage of the cohort to registers after ethical vetting.

This study is reported as per the Strengthening the Reporting of Observational Studies in Epidemiology (STROBE) guideline (S1 Checklist).

### Assessment of lipid biomarkers

TC (mmol/L), LDL-C (mmol/L), HDL-C (mmol/L), TG (mmol/L), ApoB (g/L), and ApoA-I (g/L) were selected as biomarkers for lipid metabolism. The TG/HDL-C ratio and ApoB/ApoA-I ratio was calculated accordingly. Serum levels of TC and TG were measured enzymatically, and ApoA-1 and ApoB were measured by immunoturbidimetric methods. The total coefficient of variation was <3% for TC and <5% for other lipid biomarkers. LDL-C and HDL-C levels were calculated according to the Jungner method using the following formula: LDL-C = 0.48 + 0.99 × TC − 0.23 × TG − 1.58 × ApoA-I; HDL-C = TC − 0.45 × TG − LDL-C. The Jungner formula has previously been described and validated [8,12].

### Ascertainment of AF and other conditions

The Swedish National Patient Register contains information on hospital discharge records from inpatient care regionally since 1964 and nationwide since 1987; data on specialized outpatient care was available since 2001. Information retrieved from this register includes the dates and discharge diagnoses of each hospital visit, and all discharge diagnoses were coded according to the International Classification of Diseases (ICD). The National Cause of Death Register is a complete register of all deaths in Sweden since 1952, and all underlying and contributing causes of deaths were recorded following the ICD system. In this study, both the National Patient Register and Cause of Death Register were used to identify AF (ICD-8: 427.90 and 427.92; ICD-9: 427.3; ICD-10: I48), HF (ICD-8: 427.0, 427.1; ICD-9: 402, 404, 425, 428; ICD-10: I110, I130, I132, I27, I280, I42, I43, I515, I517, I528), CHD (ICD-8: 410–414; ICD-9: 410–414; ICD-10: I20-25), stroke (ICD-8: 431–434; ICD-9: 431–434; ICD-10: I61, I63, I64), TIA (ICD-8: 435; ICD-9: 435; ICD-10: G45), diabetes (ICD-8: 250; ICD-9: 250, 251.D; ICD-10: E10, E11, E13, E14), and hypertension (ICD-8: 400–404; ICD-9: 401–405; ICD-10: I10- I13, I15).

In the current study, incident AF is the outcome of interest. Because HF and CHD often precede the onset of AF and are closely associated with dyslipidemia, we also assessed how incident HF/CHD influenced the association between lipid levels and AF. Among those who developed incident AF, incident HF or CHD cases were ascertained only if they occurred prior to, on the same date, or within 1 year after AF diagnosis. Incident HF/CHD diagnoses within 1 year after AF diagnosis were included because the pathology of HF/CHD could happen before AF but it is not uncommon for the diagnoses to come shortly after. Among those who did not develop incident AF, incident HF/CHD cases that occurred prior to death, migration, or the end of follow-up were ascertained.

### Assessment of lipid-lowering medications

Beginning in July 2005, information on prescribed medications was available for all surviving participants in the National Prescribed Drug Register, and all prescriptions were recorded using the Anatomical Therapeutic Chemical (ATC) codes. Participants who were prescribed a lipid-lowering medication (ATC code C10) and picked it up at the pharmacy any time between July 1, 2005 and the end of follow-up were considered to be using lipid-lowering medications in this study.

### Statistical analysis

The association between each lipid biomarker and incident AF was first examined in the full study sample using Cox proportional hazards regression models with attained age as the time scale. Concentrations of TC, LDL-C, HDL-C, and TG were categorized using clinical cutoffs based on consensus guidelines for dyslipidemia management [13,14]. The reference group for TC, LDL-C, HDL-C, and TG is 4.14 to 5.17, 2.59 to 3.34, 1.50 to 1.99, and 1.00 to 1.68 mmol/L, respectively. For TG/HDL-C ratio, ApoB, ApoA-I, and ApoB/ApoA-I ratio, where clinical cutoffs are lacking, quintiles were applied, and the reference point were chosen based on previous evidence for cardiovascular risk [8,10,15], which fell into the third quintile of these biomarkers. Since individuals with a history of CVD and cardiovascular risk factors at baseline have been excluded from the study population, all analyses were adjusted for baseline age, sex, and SES. To account for incident CVD that occurred during the follow-up, all analyses were additionally stratified by incident HF/CHD. To examine whether the associations between lipid levels and incident AF differ by different follow-up periods, stratified analyses were also performed by duration of follow-up, i.e., 0 to 5 years, 5 to 10 years, and ≥10 years.

Use of lipid-lowering drugs might be an important confounder in the association between lipid levels and subsequent AF. However, at baseline of this study (1985 to 1996), lipid-lowering drugs were not prescribed in Sweden or at least were still uncommon. It is therefore not likely that such drugs could have influenced the association observed during the first few years of follow-up. To investigate if and how lipid-lowering drugs influenced the association over a longer follow-up, we performed a sensitivity analysis in a subsample of participants alive and free of AF on July 1, 2005 (start of the Swedish National Prescribed Drug Register). Participants were stratified into developing incident HF/CHD or not and using lipid-lowering treatment or not. Among these subgroups, the association between each biomarker and incident AF was examined using Cox regression models, adjusting for age, sex, and SES.

There was no formal documented prospective analysis plan for the current study. However, analyses were planned prior to the examination of the data, described in ethical vetting, and no data-driven changes were made to the analyses. Clinical cutoff levels for biomarkers were used in primary analyses, and each lipid biomarker was additionally analyzed as a continuous variable in relation to incident AF using Cox regression models with restricted cubic splines. The association between each lipid marker and AF without any covariate adjustment was also examined. The associations were also analyzed separately in people aged 45 to 49, 50 to 54, and 55 to 60 years at baseline. Finally, in the analyses examining the associations of HDL-C, TG, TG/HDL-C ratio, and ApoA-I categories with incident AF, LDL-C levels were further adjusted for in addition to age, sex, and SES.

Stata/SE 16.1 (StataCorp LLC, College Station, Texas, United States of America) for Windows was used for all analysis.

## Results

A total of 65,136 individuals (36,813 men and 28,323 women) were included in the study. Baseline characteristics by sex are shown in Table 1. During the follow-up period (mean 24.2 years, standard deviation 7.5, range 0.2 to 35.9), 13,871 (21.3%) persons developed incident AF. Among these incident AF cases, 6,179 (44.6%) also developed incident HF or CHD prior to, on the same date, or within 1 year after AF diagnosis.

**Table 1:** Baseline characteristics of the study population by sex.

Characteristics	Total	Men	Women
Number of participants (%)	65,136	36,813	28,323
Age, mean (SD)	51.8 (4.5)	51.6 (4.5)	52.1 (4.5)
SES, n (%)			
Skilled/unskilled laborers	15,606 (24.0)	9,626 (26.2)	5,980 (21.1)
Employees lower level	14,373 (22.1)	4,009 (10.9)	10,364 (36.6)
Employees middle level	14,099 (21.7)	9,065 (24.6)	5,034 (17.8)
Employees higher level	12,620 (19.4)	9,357 (25.4)	3,263 (11.5)
Self-employed	2,494 (3.8)	1,799 (4.9)	695 (2.5)
Others	5,944 (9.1)	2,957 (8.0)	2,987 (10.5)
TC (mmol/L), mean (SD)	6.17 (1.13)	6.16 (1.09)	6.17 (1.17)
LDL-C (mmol/L), mean (SD)	3.96 (1.05)	4.01 (1.01)	3.88 (1.10)
HDL-C (mmol/L), mean (SD)	1.58 (0.41)	1.45 (0.37)	1.74 (0.42)
TG (mmol/L), mean (SD)	1.41 (0.82)	1.55 (0.86)	1.22 (0.72)
TG/HDL-C ratio, mean (SD)	1.04 (0.86)	1.22 (0.93)	0.81 (0.69)
ApoB (g/L), mean (SD)	1.32 (0.34)	1.37 (0.33)	1.26 (0.35)
ApoA-I (g/L), mean (SD)	1.46 (0.23)	1.40 (0.20)	1.54 (0.23)
ApoB/ApoA-I ratio, mean (SD)	0.93 (0.29)	1.00 (0.28)	0.84 (0.27)

AF, atrial fibrillation; ApoA-I, apolipoprotein A-I; ApoB, apolipoprotein B; HDL-C, high-density lipoprotein cholesterol; LDL-C, low-density lipoprotein cholesterol; SD, standard deviation; SES, socioeconomic status; TC, total cholesterol; TG, triglycerides.

**Fig 2:**
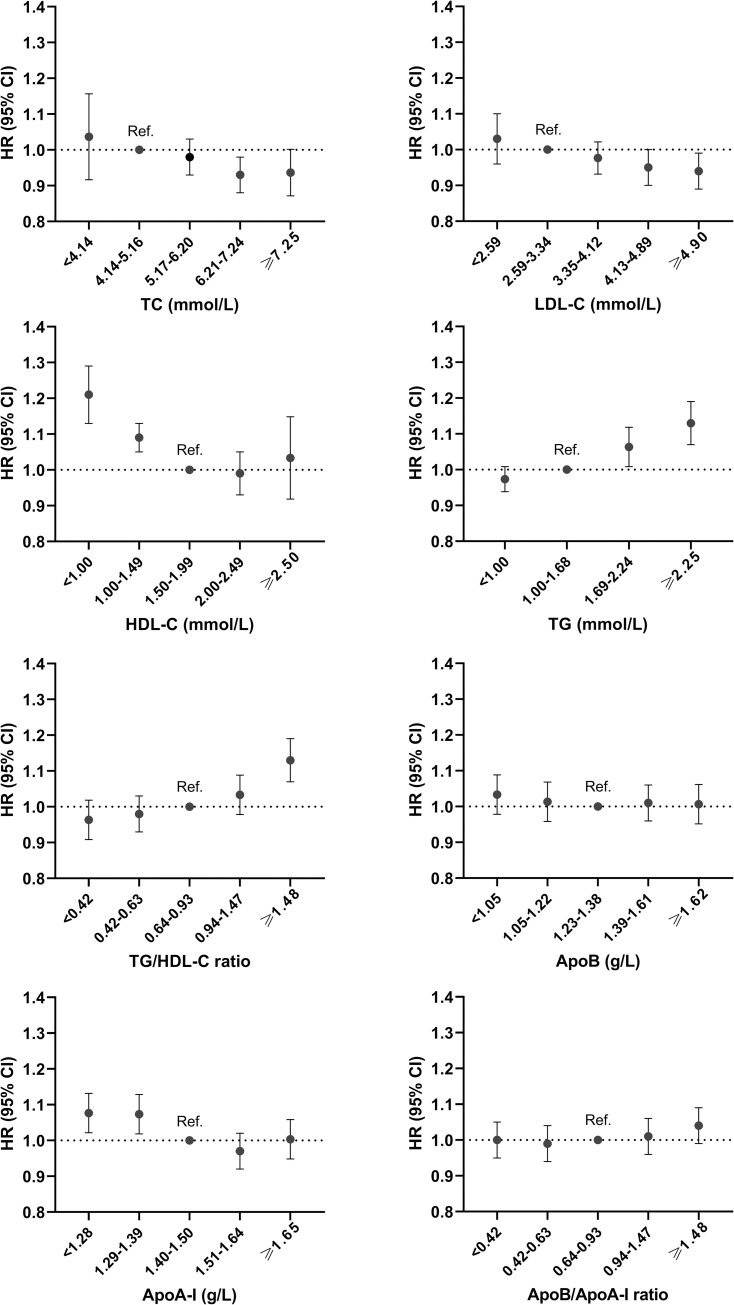
HR (95% CI) for incident AF associated with lipid biomarkers in categories. All models are adjusted for age, sex, and SES at baseline. AF, atrial fibrillation; ApoA-I, apolipoprotein A-I; ApoB, apolipoprotein B; CI, confidence interval; HDL-C, high-density lipoprotein cholesterol; HR, hazard ratio; LDL-C, low-density lipoprotein cholesterol; SES, socioeconomic status; TC, total cholesterol; TG, triglycerides.

Fig 2 shows the hazard ratios (HRs) and 95% confidence interval (CI) for AF by lipid biomarker categories after adjusting for age, sex, and SES. Over the entire follow-up, higher levels of TC and LDL-C in midlife were associated with a slightly lower risk of AF (HR ranging from 0.93 to 0.95, *p* < 0.05), compared to the reference level (4.14 to 5.17 mmol/L for TC and 2.59 to 3.34 mmol/L for LDL-C). Lower levels of HDL-C and higher levels of TG and TG/HDL-C ratio were respectively associated with a higher risk of AF (HR ranging from 1.06 to 1.21, *p* < 0.05) compared to the reference level (1.50 to 1.99 mmol/L for HDL-C, 1.00 to 1.66 mmol/L for TG, and 0.64 to 0.93 for TG/HDL-C ratio). Similar to HDL-C, low ApoA-I (<1.28 and 1.28 to 1.39 g/L) was associated with a higher risk of AF (HR ranging from 1.07 to 1.08, *p* < 0.05). With regards to ApoB and ApoB/ApoA-I ratio, neither high nor low levels were significantly associated with incident AF. Stratified analyses by age groups (45 to 49, 50 to 54, and 55 to 60 years) are shown in S1 Fig. No substantial age differences were observed in the association between lipids biomarkers and incident AF, except for high TC and HDL-C in age group 45 to 49, where high levels were not significantly associated with AF risk.

**Fig 3:**
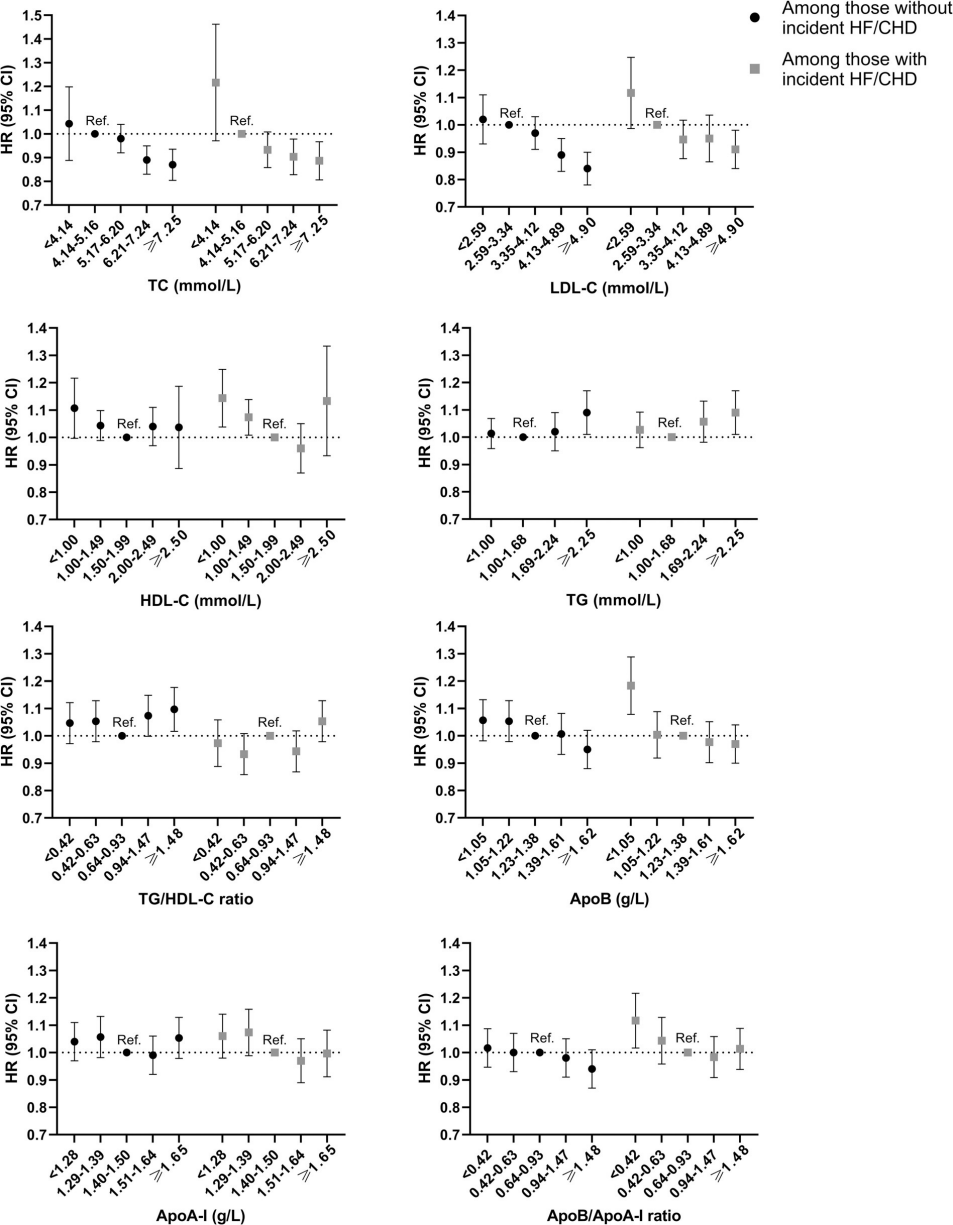
HR (95% CI) for incident AF associated with lipid biomarkers in categories, stratified by incident HF/CHD. All models are adjusted for age, sex, and SES at baseline. AF, atrial fibrillation; ApoA-I, apolipoprotein A-I; ApoB, apolipoprotein B; CHD, coronary heart disease; CI, confidence interval; HDL-C, high-density lipoprotein cholesterol; HF, heart failure; HR, hazard ratio; LDL-C, low-density lipoprotein cholesterol; SES, socioeconomic status; TC, total cholesterol; TG, triglycerides.

Stratified analyses were performed among people with or without incident HF/CHD. The absolute risk (i.e., incidence rate per 1,000 person-years) of AF were substantially higher among people who developed HF/CHD during the follow-up compared to those who did not (S2 Fig). With regards to relative risk, high TC and LDL-C levels were consistently associated with lower risk of AF, both among people with incident HF/CHD and among those without (p for interaction >0.1) (Fig 3). Likewise, low HDL-C and high TG levels were associated with a higher risk of AF, both among people with and without incident HF/CHD (p for interaction >0.1). ApoB and ApoB/ApoA-I ratio categories were not associated with incident AF in either subgroup, except for low level of ApoB (<1.05 g/L) and ApoB/ApoA-I ratio (<0.42) that was associated with a higher risk of AF only among those who developed incident HF/CHD (p for interaction = 0.019 and 0.009, respectively). When analyzed as continuous variables using cubic splines, the associations between each biomarker and incident AF were similar compared to when lipid biomarkers were analyzed categorically (S3 and S4 Figs).

**Fig 4:**
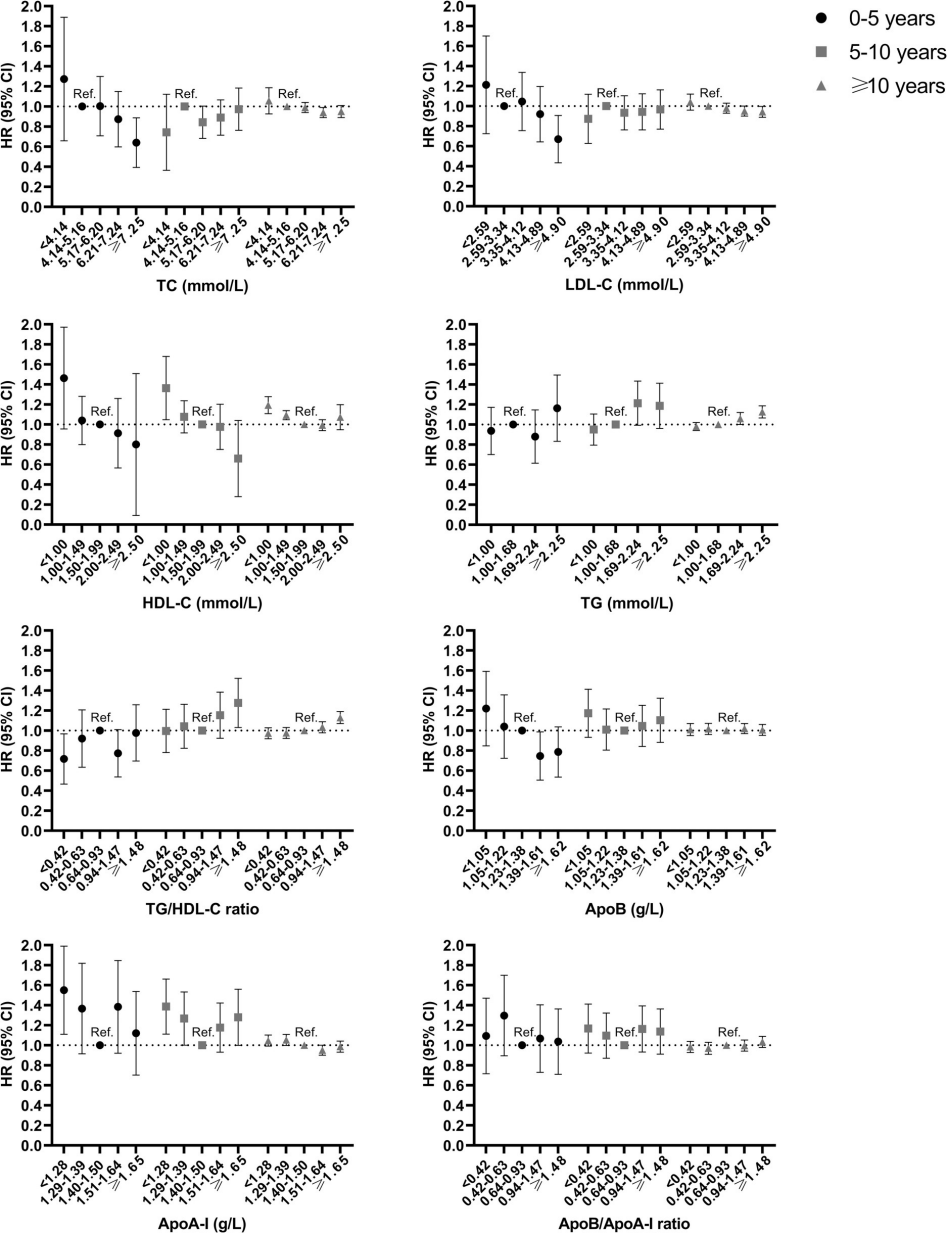
HR (95% CI) for incident AF associated with lipid biomarkers in categories, stratified by periods of follow-up (i.e., 0–5 years, 5–10 years, and ≥10 years). All models are adjusted for age, sex, and SES at baseline. AF, atrial fibrillation; ApoA-I, apolipoprotein A-I; ApoB, apolipoprotein B; CI, confidence interval; HDL-C, high-density lipoprotein cholesterol; HR, hazard ratio; LDL-C, low-density lipoprotein cholesterol; SES, socioeconomic status; TC, total cholesterol; TG, triglycerides.

All analyses were further stratified by durations of follow-up to examine whether the observed associations differed by time since biomarker measurement (Fig 4). High levels of TC (≥7.25 mmol/L) and LDL-C (≥4.90 mmol/L) were respectively associated with a lower risk of AF during the first 5 years of follow-up (HR = 0.61, 95% CI: 0.41 to 0.99, *p* = 0.013; HR = 0.64, 95% CI: 0.45 to 0.92, *p* = 0.016), but were not associated with incident AF during 5 to 10 years of follow-up (HR = 0.96, 95% CI: 0.77 to 1.19, *p* > 0.05; HR = 0.95, 95% CI: 0.78 to 1.17, *p* > 0.05), and only very weakly associated with a lower risk of AF from 10 years onward (HR = 0.94, 95% CI: 0.89 to 1.00, *p* = 0.038; HR = 0.95, 95% CI: 0.89 to 1.01, *p* > 0.05). On the other hand, low HDL-C (<1.00 mmol/L) and high TG/HDL-C ratio (≥1.48) were respectively associated with a higher risk of AF both during 5 to 10 years of follow-up and from 10 years onward (HR ranging from 1.13 [95% CI: 1.07 to 1.19, *p* < 0.001] to 1.34 [95% CI: 1.06 to 1.69, *p* = 0.015]). The results for ApoA-I were similar to that of HDL-C, where low ApoA-I (<1.28 g/L) was associated with a higher risk of AF over the follow-up (HR = 1.53, 95% CI: 1.12 to 2.00, *p* = 0.007; HR = 1.37, 95% CI: 1.12 to 1.67, *p* = 0.002; HR = 1.05, 95% CI: 1.00 to 1.10, *p* = 0.065). The results for ApoB were similar to that of LDL-C. Neither low nor high ApoB/ApoA-I ratio was associated with incident AF in any of the follow-up periods.

Unadjusted comparisons for the above analyses are shown in S5–S7 Figs, and the crude HRs were not substantially different from the models adjusted for age, sex, and SES in Figs 2–4. Additionally, adjusting for LDL-C levels in the analyses for HDL-C, TG, TG/HDL-C ratio, and ApoA-I did not alter their association with incident AF (S8 and S9 Figs).

The sensitivity analyses included 56,493 participants alive and free of AF by July 1, 2005, and for whom data on use of medications were available from this date onward. The lipid biomarker levels at baseline were very similar between the full cohort and the subsample (S1 Table). All analyses for the association between lipid categories and incident AF in this subsample were stratified by developing incident HF/CHD or not and using lipid-lowering drugs or not. The start of follow-up in this subsample was set at July 1, 2005, more than 10 years after baseline blood measurement (corresponding to follow-up period ≥10 years in the main analyses). Therefore, the IR of AF in this subsample was overall higher than that in the full study sample (S2 Table). Among people with incident HF/CHD, the IR of AF was substantially lower in those who used lipid-lowering drugs than in those who did not. Among people without incident HF/CHD, the IR of AF did not differ substantially by treatment status. High levels of TC, LDL-C, ApoB, and ApoB/ApoA-I ratio were not associated with incident AF in any of the subgroup analyses (p for interaction >0.1), except for ApoB/ApoA-1 ≥0.94 in untreated participants with incident HF/CHD (HR = 1.27, 95% CI: 1.10 to 1.45) (p for interaction = 0.003).

## Discussion

In this large population-based study with more than 65,000 cardiovascular healthy individuals at baseline, aged 45 to 60 years, and followed for up to 35 years, a lower risk of AF in association with high TC and LDL-C levels was observed. However, these associations were only present during the first 5 years after blood measurement. For HDL-C and ApoA-I, low levels were associated with an increased risk of AF, and this did not change with time of follow-up. The association between a high TG and TG/HDL-C ratio and higher risk of AF also persisted over the follow-up. ApoB/ApoA-I ratio was not associated with AF, except for elevated ratio (≥0.94) in untreated participants with incident HF/CHD. The association between lipids and AF does not seem to differ by co-occurring HF/CHD.

Our findings of a protective effect of higher TC and LDL-C are consistent with previous smaller studies with shorter follow-up time, such as the Atherosclerosis Risk in Communities study (*n* = 13,969), which reported an association between high TC and LDL-C levels and lower incidence of AF over a median follow-up of 18.7 years [3]. One recent systematic review and meta-analysis using pooled data from >4 million participants reported a 5% decreased risk of AF associated with every 1 mmol/L increment in TC or LDL-C levels at study baseline [16]. In this study, we explored whether these associations differ by co-occurring CVD and found that even though individuals with incident HF/CHD had higher absolute risk of AF, the relative risk of AF with high TC and LDL-C was the same in individuals with and without HF/CHD. Unlike previous studies, the current study also examined whether such associations differ by time of follow-up, and found that the association was only present during the first 5 years after lipid measurement. One explanation for this can be that cholesterol levels change over time, for example, individuals with high TC or LDL-C at baseline could have started lipid-lowering treatment during the follow-up. However, the sensitivity analyses did not provide evidence that use of lipid-lowering drugs during the follow-up explains the lack of associations 10 years after biomarker measurement. Another explanation can be that TC and LDL-C changed for other reasons and the baseline measurement is no longer precise and becomes less predictive of AF in time periods longer than 5 years. It can also be that high cholesterols in midlife may only have a short-term effect in reducing the risk of AF and becomes less relevant in determining risk of AF in older ages (e.g., ≥70 years). The mechanisms of how high TC and LDL-C are associated with a lower risk of AF remain speculative. It was shown in in vitro studies that cholesterol as the main components of cell membranes can modulate the function of some ion channels that are involved in the initiation of AF [17,18].

To our knowledge, our study is the first to examine the longitudinal relationship between apolipoprotein measures and incident AF. The ApoB level represents the total number of atherogenic particles in blood including cholesterol- and TG-containing lipoprotein particles (very low-density lipoprotein, intermediate-density lipoprotein, and LDL). ApoB/ApoA-I ratio has been shown in many reports to be a more informative and robust measure in predicting CHD than other lipid fractions [8,9]. In our study, ApoB and ApoB/ApoA-I ratio was not associated with incident AF, except for low ApoB (<1.05 g/L) and ApoB/ApoA-I ratio (<0.42) that was associated with higher AF risk among those who developed incident HF/CHD. One explanation could be that they developed AF on the basis of HF/CHD or other concomitant diseases associated with low cholesterol, such as cancer [19,20]. In the sensitivity analyses, elevated ApoB/ApoA-I ratio (≥0.94) was associated with a higher AF risk in individuals with incident HF/CHD but untreated with lipid-lowering drugs. However, since many stratified analyses were performed, it could be due to chance that statistically significant results were found only in these subgroups. Nevertheless, these findings suggest that part of the etiology of AF is different from that of atherosclerotic heart diseases, and a poor atherogenic to anti-atherogenic balance may not serve as a basic mechanism behind the development of AF. Further studies are needed to replicate our results.

Only a few studies have examined the association of TG and HDL-C with AF that mostly reported no association. One study using data from 2 community-based cohorts found a higher risk of AF associated with higher TG levels [21], and 2 other studies reported a higher risk of AF associated with lower HDL-C levels [6,22]. The discrepancy in the results is likely due to methodological differences, including varying study population characteristics, baseline age, and follow-up time [23]. Our study showed that lower levels of HDL-C are associated with a higher risk of AF not only in the short term but also 10 years after blood measurement. Results for ApoA-I were generally similar to that of HDL-C. Inflammation has been proposed to be a key etiologic factor in the development of AF [24,25], and HDL-C and ApoA-I have important anti-inflammatory and antioxidant properties. Low HDL-C and ApoA-I levels in midlife may reflect underlying chronic inflammation over the lifespan that further elevates the risk of AF. In addition, low HDL-C is also an important component in metabolic syndrome, and metabolic syndrome has been associated with the development of AF. In this study, a higher risk of AF was observed among people with high TG/HDL-C ratio, which in turn is associated with insulin resistance [26], indicating that metabolic syndrome plays a role in the onset of AF [27–29]. Metabolic syndrome can also contribute to atrial stretch and dilation via mechanical stress, resulting in structural and electrophysiological remodeling predisposing to AF [28].

This study has several strengths. To our knowledge, it is by far the largest study of blood lipid levels and AF in terms of sample size and follow-up time, and we were able to perform subgroup analyses by co-occurring CVD and durations of follow-up to provide a deeper understanding of lipids and AF risk. Given the high quality of Swedish national registers, almost no participants were lost to follow-up. All biomarkers were analyzed in the same lab with well-documented methodologies. Furthermore, the association with incident AF was analyzed for not only conventional lipids but also apolipoproteins, which few studies have examined. Nevertheless, the current study has important limitations. First, between 1987 and 2000, AF was identified from hospital diagnosis in the patient register that included data from inpatient care throughout the study period. From 2001, data from specialized outpatient care became available. Therefore, it is likely that the current study missed some AF cases before 2001 when outpatient data were not available and some asymptomatic cases throughout the study period. However, this potential misclassification of AF is most likely not related to the baseline lipid levels and therefore could have resulted in a dilution of the association. Second, it is also possible that some conditions, for example, hypertension, could not be fully captured by the patient register. Although people with a history of diagnosed CVD and cardiovascular risk factors have been excluded from baseline, the baseline sample could therefore still have included some individuals with undiagnosed hypertension. This could have led to some residual confounding. Third, we were also not able to account for certain lifestyle factors such as smoking, alcohol consumption, and physical activity. This is indeed a limitation, even though the biomarkers can be seen, at least partly, as the result of such lifestyle factors. Nevertheless, the observed association could be subject to residual confounding. Finally, single lipid measurement at baseline may not accurately reflect lipid variability over the very long follow-up in the current study. It has been shown that HDL-C levels tend to be more stable over time as compared to TC and LDL-C [30,31], and this study indeed found a stable association between low HDL and higher AF risk throughout the study period. Nevertheless, future studies should explore how longitudinal changes in lipid levels may predict the risk of AF.

## Conclusions

In this large population-based study, a reduced risk of AF was found in association with high TC and LDL-C up to 5 years after blood measurement and not thereafter. On the contrary, low HDL-C and ApoA-I and high TG/HDL-C ratio was consistently associated with a higher risk of AF over 3 decades of follow-up. A poor atherogenic and anti-atherogenic balance, as indicated by a high ApoB/ApoA-I ratio, is not associated with a higher AF risk.

## Supporting information

S1 Checklist STROBE Statement—Checklist of items that should be included in reports of cohort studies. (DOCX)

S1 Fig HRs (95% CI) of AF associated with lipid biomarker categories, stratified by age groups at baseline. All models were adjusted for age, sex, and SES. AF, atrial fibrillation; CI, confidence interval; HR, hazard ratio; SES, socioeconomic status.
(TIF)

S2 Fig Incidence rate (95% CI) of AF per 1,000 person-years associated with lipid biomarker categories, stratified by incident HF /CHD. AF, atrial fibrillation; CHD, coronary heart disease; CI, confidence interval; HF, heart failure.
(TIF)

S3 Fig HRs (95% CI) of AF associated with lipid biomarkers analyzed as continuous variables. Solid lines represent HRs and dotted lines represent distribution of each biomarker in the analytical sample. All models were adjusted for age, sex, and SES. AF, atrial fibrillation; CI, confidence interval; HR, hazard ratio; SES, socioeconomic status.
(TIF)

S4 Fig HRs (95% CI) of AF associated with lipid biomarkers analyzed as continuous variables, stratified by incident HF/CHD. Solid lines represent HRs and dotted lines represent the distribution of each biomarker in the analytical sample. All models were adjusted for age, sex, and SES. AF, atrial fibrillation; CHD, coronary heart disease; CI, confidence interval; HF, heart failure; HR, hazard ratio; SES, socioeconomic status.
(TIF)

S5 Fig Unadjusted HR (95% CI) of AF associated with lipid biomarker categories. AF, atrial fibrillation; CI, confidence interval; HR, hazard ratio.
(TIF)

S6 Fig Unadjusted HR (95% CI) for incident AF associated with lipid biomarkers in categories, stratified by incident HF/CHD. AF, atrial fibrillation; CHD, coronary heart disease; CI, confidence interval; HF, heart failure; HR, hazard ratio.
(TIF)

S7 Fig Unadjusted HR (95% CI) for incident AF associated with lipid biomarkers in categories, stratified by periods of follow-up (i.e., 0–5 years, 5–10 years, and ≥10 years). AF, atrial fibrillation; CI, confidence interval; HR, hazard ratio.
(TIF)

S8 Fig HRs (95% CI) of AF associated with HDL-C, TG, TG/HDL-C ratio, and ApoA-I categories, stratified by incident /CHD. All models were adjusted for age, sex, SES, and LDL-C levels. AF, atrial fibrillation; ApoA-I, apolipoprotein A-I; CHD, coronary heart disease; CI, confidence interval; HDL-C, high-density lipoprotein cholesterol; HF, heart failure; HR, hazard ratio; LDL-C, low-density lipoprotein cholesterol; SES, socioeconomic status; TG, triglyceride.
(TIF)

S9 Fig HRs (95% CI) of AF associated with HDL-C, TG, TG/HDL-C ratio, and ApoA-I categories, stratified by periods of follow-up (i.e., 0–5 years, 5–10 years, and ≥10 years). All models were adjusted for age, sex, SES, and LDL-C levels. AF, atrial fibrillation; ApoA-I, apolipoprotein A-I; CI, confidence interval; HDL-C, high-density lipoprotein cholesterol; HR, hazard ratio; LDL-C, low-density lipoprotein cholesterol; SES, socioeconomic status; TG, triglyceride.
(TIF)

S1 Table Participant characteristics comparing the full study sample and the subsample. (DOCX)

S2 Table Incidence rate and HRs (95% CI) for incident AF associated with TC, LDL-C, ApoB, and ApoB/ApoA-I categories stratified by use of lipid-lowering drugs and incident HF/CHD (subsample *n* = 56493). ApoA-I, apolipoprotein A-I; ApoB, apolipoprotein B; CHD, coronary heart disease; CI, confidence interval; HF, heart failure; HR, hazard ratio; LDL-C, low-density lipoprotein cholesterol; TC, total cholesterol.
(DOCX)

## Abbreviations

AF: atrial fibrillation
AMORIS: Apolipoprotein-Related Mortality Risk
ApoA-I: apolipoprotein A-I
ApoB: apolipoprotein B
CHD: coronary heart disease
CI: confidence interval
CVD: cardiovascular disease
HDL-C: high-density lipoprotein cholesterol
HF: heart failure
HR: hazard ratio
LDL-C: low-density lipoprotein cholesterol
SES: socioeconomic status
TC: total cholesterol
TG: triglyceride
TIA: transient ischemic attack
