# Supplementary figures and images for: Lipid levels in midlife and risk of atrial fibrillation over 3 decades—Experience from the Swedish AMORIS cohort: A cohort study
Source: PLoS Med. 2022 Aug 11;19(8):e1004044. doi: 10.1371/journal.pmed.1004044 (PMC9371362; doi:10.1371/journal.pmed.1004044)

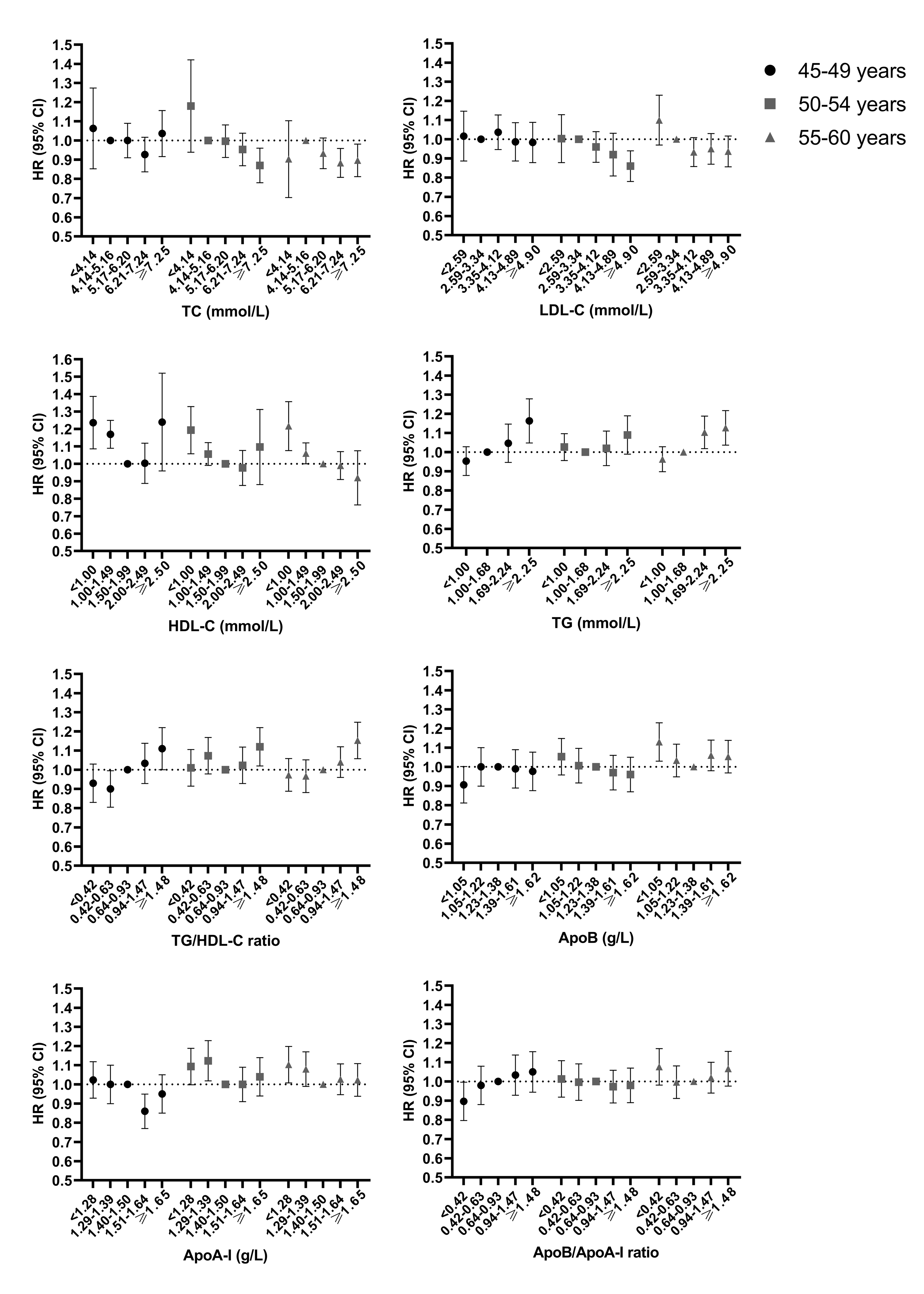

Supplement: S1 Fig — All models were adjusted for age, sex, and SES. AF, atrial fibrillation; CI, confidence interval; HR, hazard ratio; SES, socioeconomic status. (TIF) [file pmed.1004044.s002.tif]

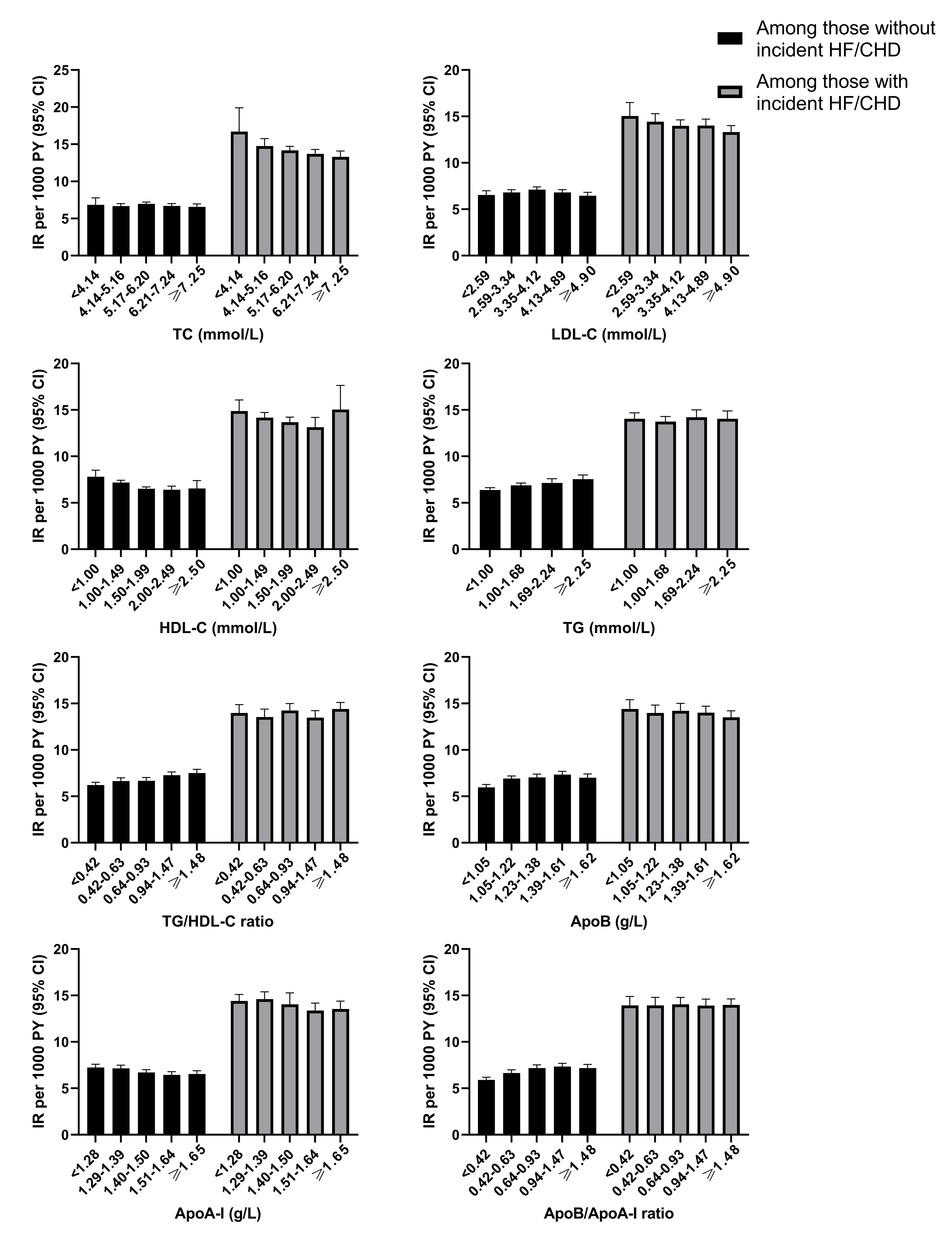

Supplement: S2 Fig — AF, atrial fibrillation; CHD, coronary heart disease; CI, confidence interval; HF, heart failure. (TIF) [file pmed.1004044.s003.tif]

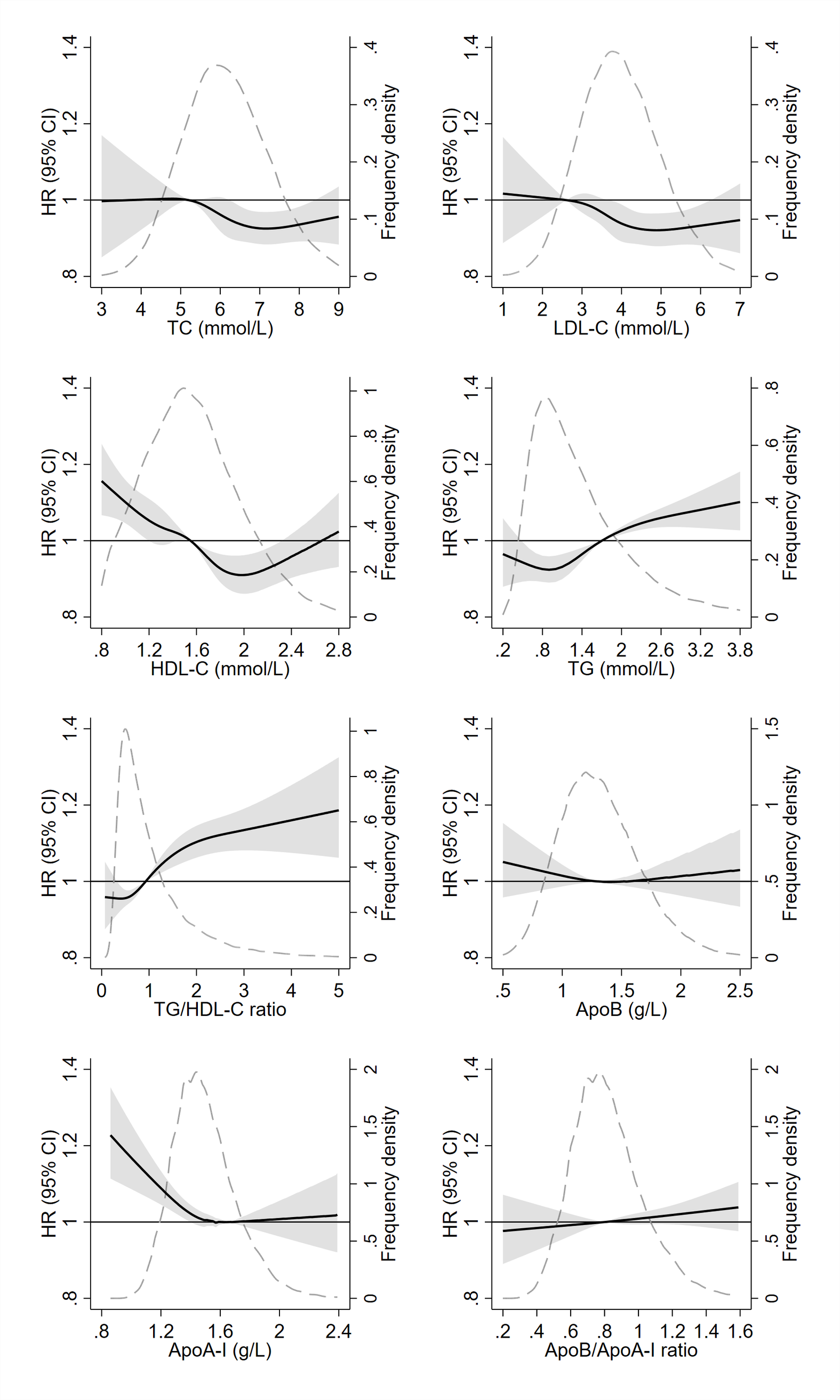

Supplement: S3 Fig — Solid lines represent HRs and dotted lines represent distribution of each biomarker in the analytical sample. All models were adjusted for age, sex, and SES. AF, atrial fibrillation; CI, confidence interval; HR, hazard ratio; SES, socioeconomic status. (TIF) [file pmed.1004044.s004.tif]

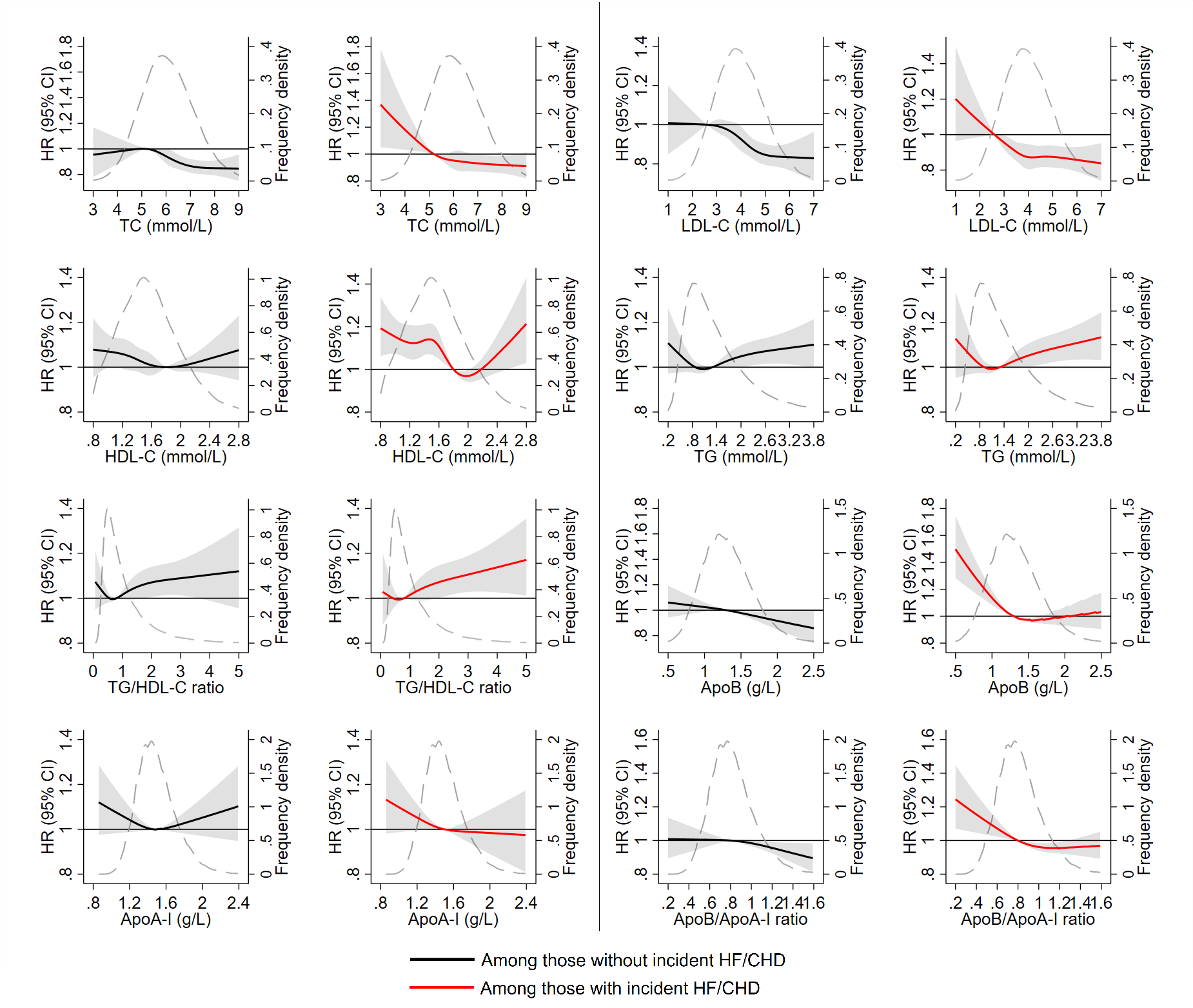

Supplement: S4 Fig — Solid lines represent HRs and dotted lines represent the distribution of each biomarker in the analytical sample. All models were adjusted for age, sex, and SES. AF, atrial fibrillation; CHD, coronary heart disease; CI, confidence interval; HF, heart failure; HR, hazard ratio; SES, socioeconomic status. (TIF) [file pmed.1004044.s005.tif]

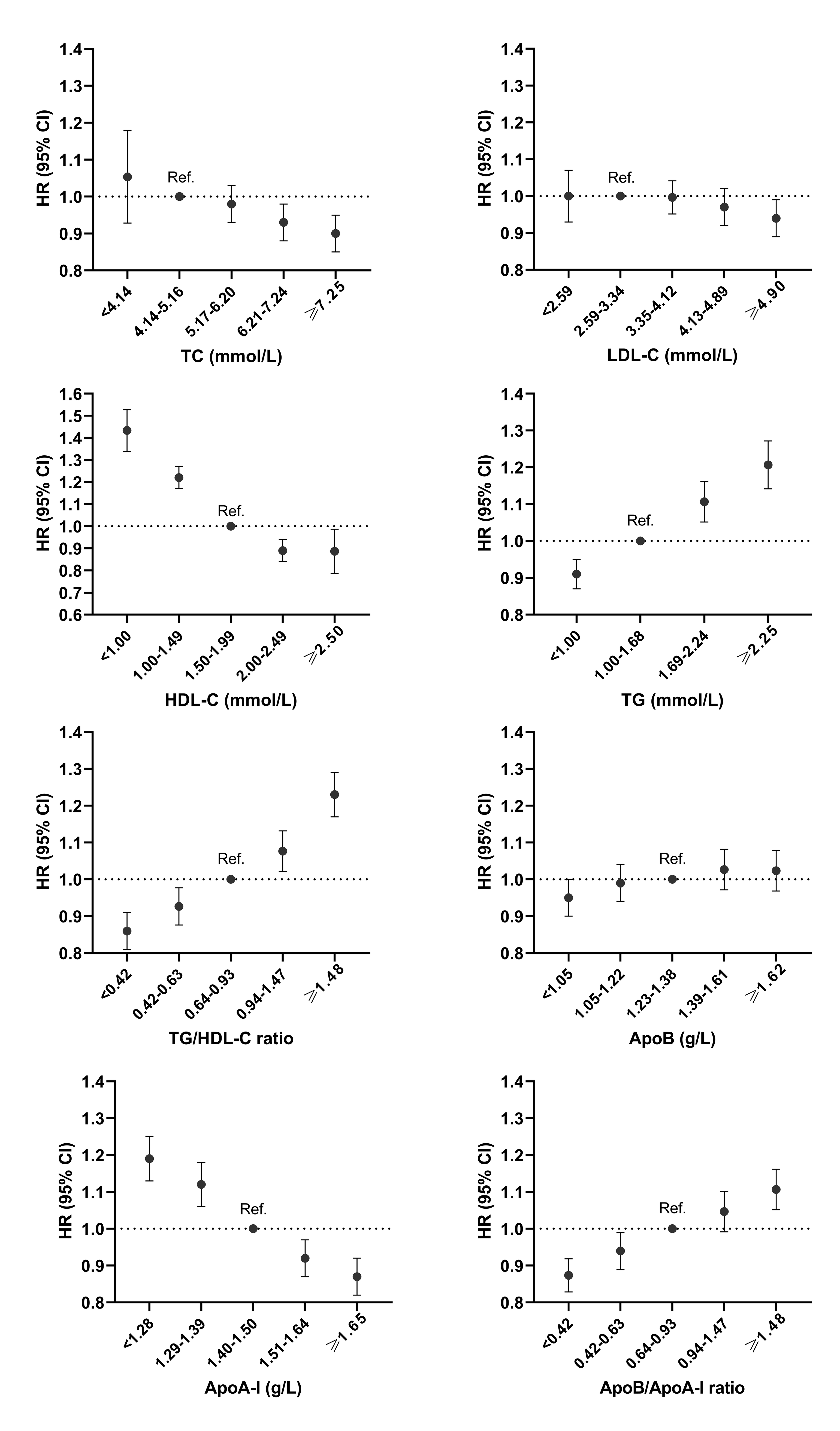

Supplement: S5 Fig — AF, atrial fibrillation; CI, confidence interval; HR, hazard ratio. (TIF) [file pmed.1004044.s006.tif]

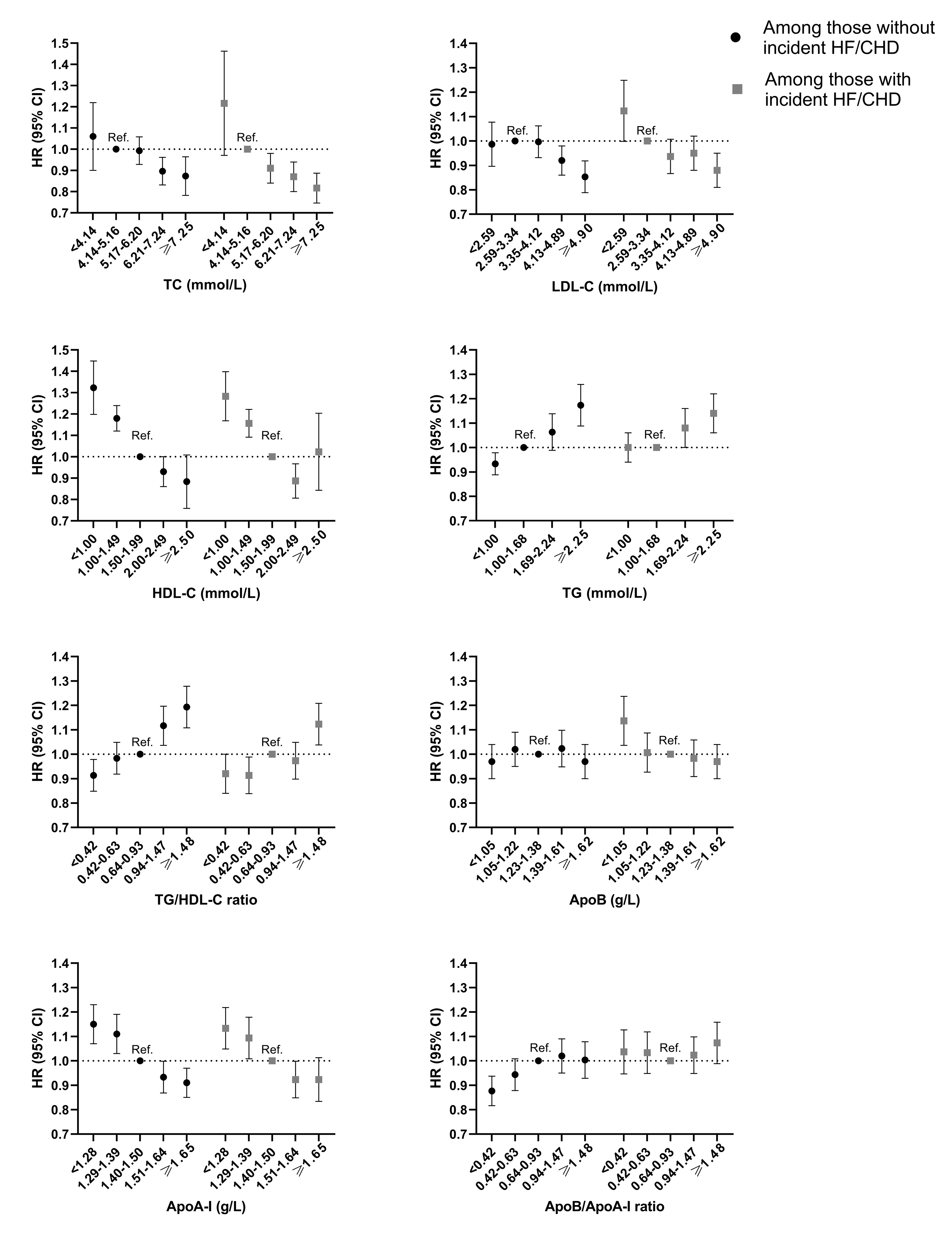

Supplement: S6 Fig — AF, atrial fibrillation; CHD, coronary heart disease; CI, confidence interval; HF, heart failure; HR, hazard ratio. (TIF) [file pmed.1004044.s007.tif]

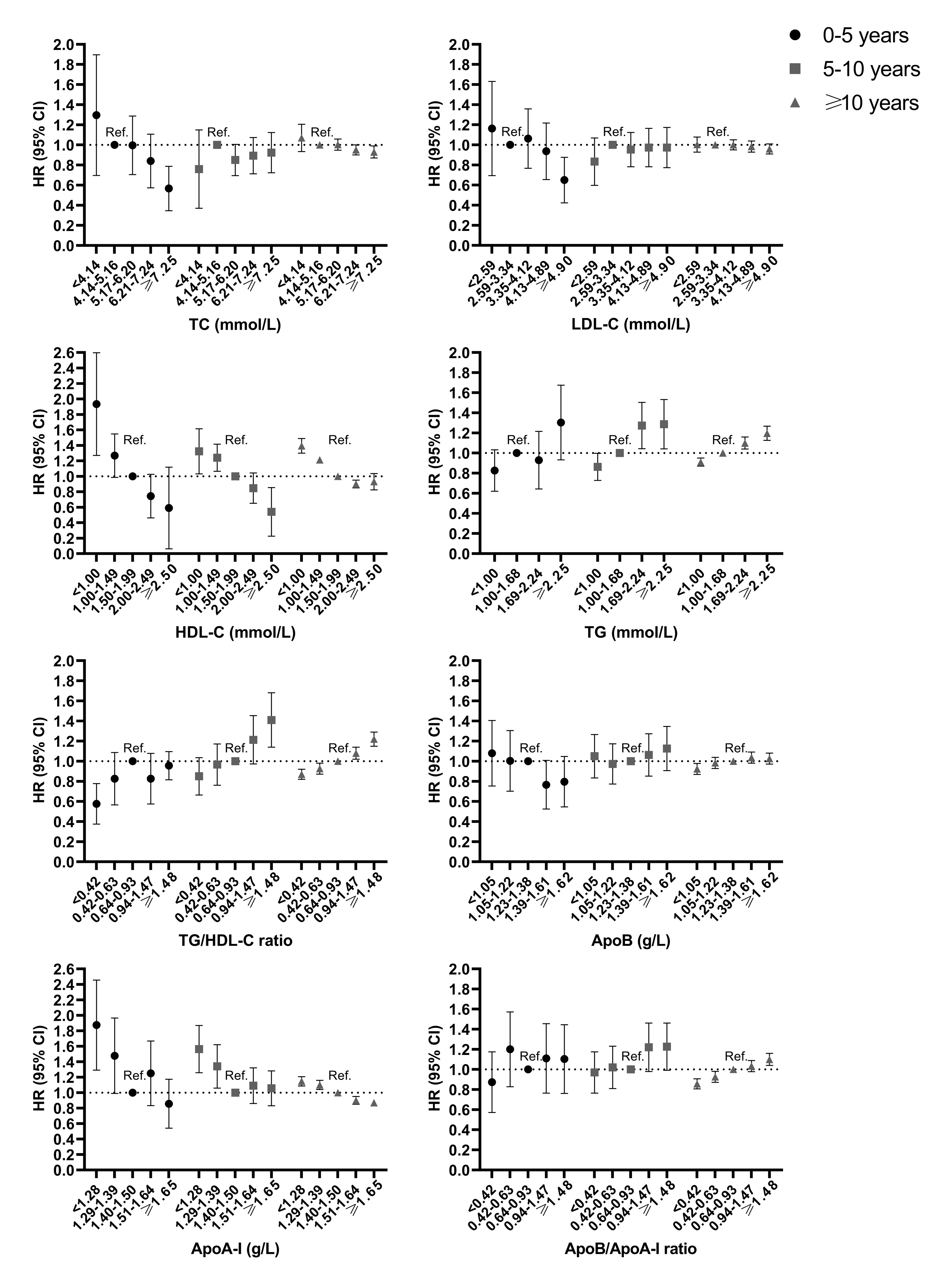

Supplement: S7 Fig — AF, atrial fibrillation; CI, confidence interval; HR, hazard ratio. (TIF) [file pmed.1004044.s008.tif]

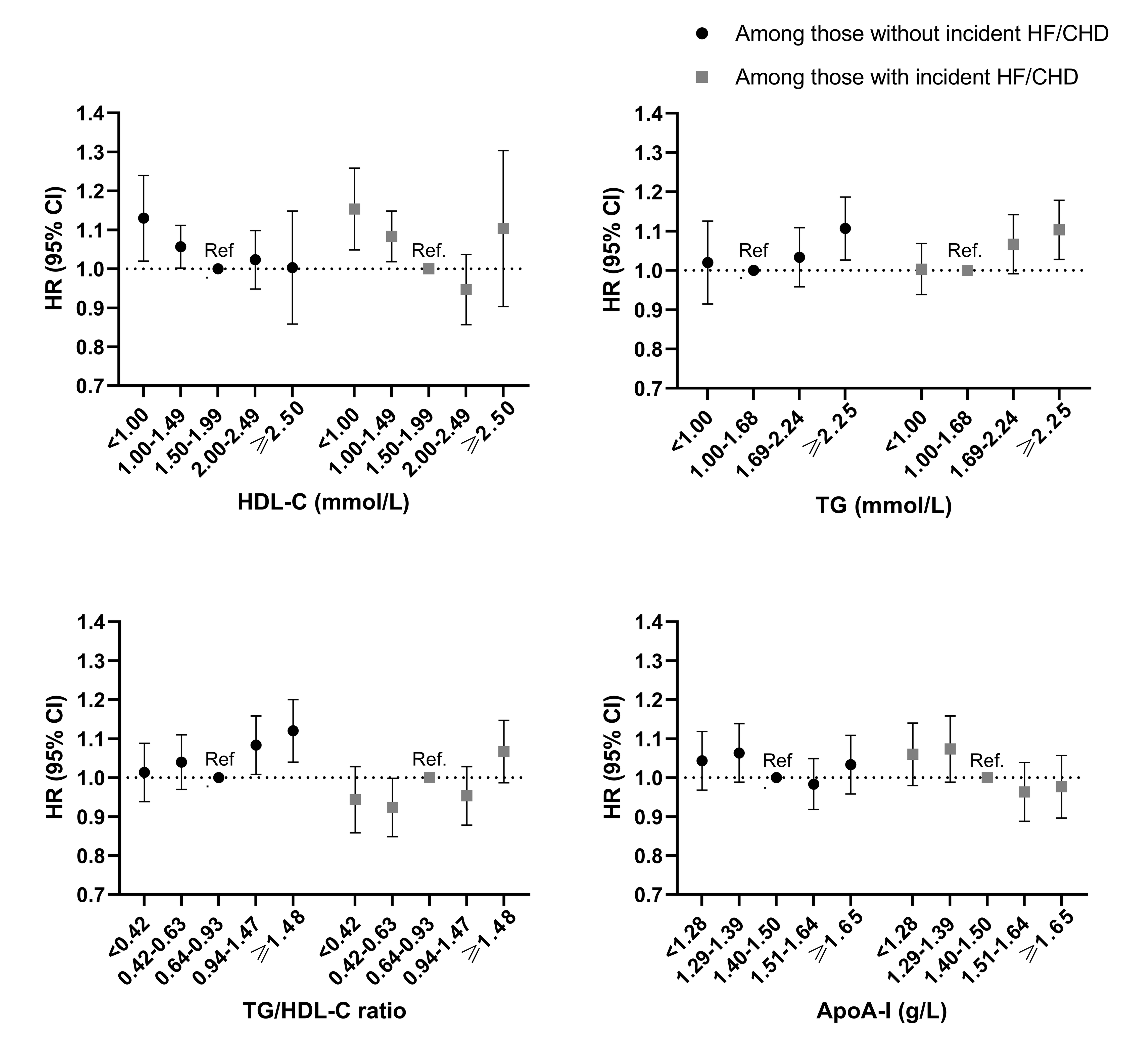

Supplement: S8 Fig — All models were adjusted for age, sex, SES, and LDL-C levels. AF, atrial fibrillation; ApoA-I, apolipoprotein A-I; CHD, coronary heart disease; CI, confidence interval; HDL-C, high-density lipoprotein cholesterol; HF, heart failure; HR, hazard ratio; LDL-C, low-density lipoprotein cholesterol; SES, socioeconomic status; TG, triglyceride. (TIF) [file pmed.1004044.s009.tif]

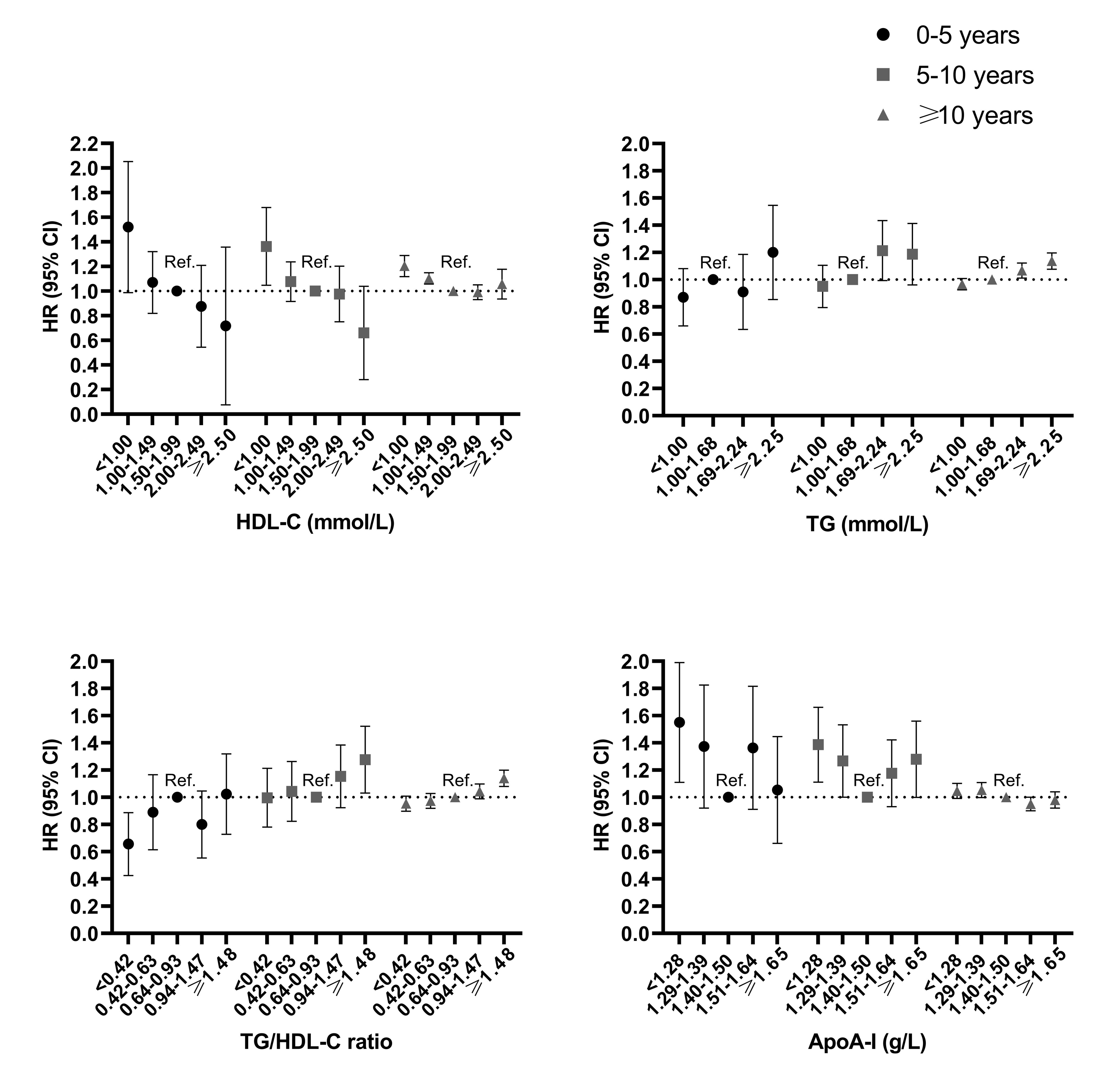

Supplement: S9 Fig — All models were adjusted for age, sex, SES, and LDL-C levels. AF, atrial fibrillation; ApoA-I, apolipoprotein A-I; CI, confidence interval; HDL-C, high-density lipoprotein cholesterol; HR, hazard ratio; LDL-C, low-density lipoprotein cholesterol; SES, socioeconomic status; TG, triglyceride. (TIF) [file pmed.1004044.s010.tif]
