# Supplementary material for: Lipid levels in midlife and risk of atrial fibrillation over 3 decades—Experience from the Swedish AMORIS cohort: A cohort study
Source: PLoS Med. 2022 Aug 11;19(8):e1004044. doi: 10.1371/journal.pmed.1004044 (PMC9371362; doi:10.1371/journal.pmed.1004044)
Supplement: S1 Table — (DOCX) [file pmed.1004044.s011.docx]

**S1 Table. Participant characteristics comparing the full study sample and the subsample.**

| **Characteristics** | **Full sample** | **Sub-sample** |
| --- | --- | --- |
| *At baseline blood measurement* |  |  |
| Number of subjects | 65136 | 56493 |
| Age, mean (SD) | 51.8 (4.5) | 51.6 (4.5) |
| Female sex, n (%) | 28323 (43.5) | 25460 (45.1) |
| Socio-economic index, n (%) |  |  |
| Skilled/unskilled laborers | 15606 (24.0) | 13198 (23.4) |
| Employees lower level | 14373 (22.1) | 12655 (22.4) |
| Employees middle level | 14099 (21.7) | 12378 (21.9) |
| Employees higher level | 12620 (19.4) | 11120 (19.7) |
| Self-employed | 2494 (3.8) | 2137 (3.8) |
| Others | 5944 (9.1) | 5005 (8.9) |
| TC (mmol/L), mean (SD) | 6.17 (1.13) | 6.16 (1.12) |
| LDL-C (mmol/L), mean (SD) | 3.96 (1.05) | 3.95 (1.04) |
| HDL-C (mmol/L), mean (SD) | 1.58 (0.41) | 1.58 (0.41) |
| TG (mmol/L), mean (SD) | 1.41 (0.82) | 1.39 (0.82) |
| TG/HDL-C ratio, mean (SD) | 1.04 (0.86) | 1.03 (0.85) |
| ApoB (g/L), mean (SD) | 1.32 (0.34) | 1.32 (0.35) |
| ApoA-I (g/L), mean (SD) | 1.46 (0.23) | 1.46 (0.22) |
| ApoB/ApoA-I ratio, mean (SD) | 0.93 (0.29) | 0.92 (0.29) |
| *Over follow-up* |  |  |
| Use of lipid-lowering drugs^*^ | - | 31073 (55.0) |
| Follow-up time^†^ (years), mean (SD) | 24.2 (7.5) | 12.5 (4.5) |
| Incident AF, n (%) | 13871 (21.3) | 10880 (19.3) |

^*^Information on use of lipid-lowering drugs is available from July 2005 onward in the subsample.

^†^Start of follow-up in Cox regressions is the date of baseline blood measurements for the full study sample and July 1^st^, 2005 for the subsample.

AF = atrial fibrillation; ApoA-I = apolipoprotein A-I; ApoB = apolipoprotein B; HDL-C = high-density lipoprotein cholesterol; LDL-C = low-density lipoprotein cholesterol; SD = standard deviation; TC= total cholesterol; TG = triglycerides
