# Supplementary material for: Lipid levels in midlife and risk of atrial fibrillation over 3 decades—Experience from the Swedish AMORIS cohort: A cohort study
Source: PLoS Med. 2022 Aug 11;19(8):e1004044. doi: 10.1371/journal.pmed.1004044 (PMC9371362; doi:10.1371/journal.pmed.1004044)
Supplement: S2 Table — ApoA-I, apolipoprotein A-I; ApoB, apolipoprotein B; CHD, coronary heart disease; CI, confidence interval; HF, heart failure; HR, hazard ratio; LDL-C, low-density lipoprotein cholesterol; TC, total cholesterol. (DOCX) [file pmed.1004044.s012.docx]

**S2 Table. Incidence rate and hazard ratios (95% confidence interval) for incident atrial fibrillation associated with TC, LDL-C, ApoB, and ApoB/ApoA-I categories stratified by use of lipid-lowering drugs and incident heart failure/coronary heart disease (subsample n=56493)**

|  | No. of subjects | No. of cases | IR per 1000 PY (95% CI) | HR^*^ (95% CI) |
| --- | --- | --- | --- | --- |
| **Among those who developed incident HF/CHD** |  |  |  |  |
| No LLD treatment |  |  |  |  |
| TC <5.17 mmol/L | 959 | 391 | 43.9 (39.8-48.5) | 1.00 (ref.) |
| TC ≥5.17 mmol/L | 3064 | 1216 | 45.9 (43.4-48.5) | 1.02 (0.91-1.15) |
| LLD treatment |  |  |  |  |
| TC <5.17 mmol/L | 1237 | 360 | 22.9 (20.6-25.4) | 1.00 (ref.) |
| TC ≥5.17 mmol/L | 10139 | 2843 | 23.1 (22.3-23.9) | 0.99 (0.89-1.11) |
| No LLD treatment |  |  |  |  |
| LDL-C <3.35 mmol/L | 1531 | 616 | 43.3 (40.1-46.9) | 1.00 (ref.) |
| LDL-C ≥3.35 mmol/L | 2492 | 991 | 46.8 (44.0-49.8) | 1.04 (0.94-1.16) |
| LLD treatment |  |  |  |  |
| LDL-C <3.35 mmol/L | 2082 | 593 | 22.6 (20.9-24.5) | 1.00 (ref.) |
| LDL-C ≥3.35 mmol/L | 9294 | 2610 | 23.2 (22.3-24.1) | 1.02 (0.93-1.11) |
| No LLD treatment |  |  |  |  |
| ApoB <1.39 g/L | 3504 | 1410 | 44.9 (42.7-47.3) | 1.00 (ref.) |
| ApoB ≥1.39 g/L | 519 | 197 | 49.3 (42.9-56.7) | 1.06 (0.91-1.23) |
| LLD treatment |  |  |  |  |
| ApoB <1.39 g/L | 8057 | 2279 | 22.8 (21.9-23.7) | 1.00 (ref.) |
| ApoB ≥1.39 g/L | 3319 | 924 | 23.7 (22.3-25.3) | 1.01 (0.94-1.09) |
| No LLD treatment |  |  |  |  |
| ApoB/ApoA-I ratio <0.94 | 3448 | 1357 | 43.7 (41.4-46.5) | 1.00 (ref.) |
| ApoB/ApoA-I ratio ≥0.94 | 575 | 250 | 58.3 (51.5-65.9) | 1.27 (1.10-1.45) |
| LLD treatment |  |  |  |  |
| ApoB/ApoA-I ratio <0.94 | 7807 | 2202 | 22.8 (21.8-23.7) | 1.00 (ref.) |
| ApoB/ApoA-I ratio ≥0.94 | 3569 | 1001 | 23.8 (22.3-25.3) | 1.02 (0.95-1.10) |
| **Among those without incident HF/CHD** |  |  |  |  |
| No LLD treatment |  |  |  |  |
| TC <5.17 mmol/L | 5990 | 880 | 11.4 (10.6-12.2) | 1.00 (ref.) |
| TC ≥5.17 mmol/L | 15406 | 2452 | 13.0 (12.4-13.5) | 1.01 (0.94-1.10) |
| LLD treatment |  |  |  |  |
| TC <5.17 mmol/L | 2035 | 259 | 9.1 (8.1-10.2) | 1.00 (ref.) |
| TC ≥5.17 mmol/L | 17663 | 2479 | 10.4 (10.0-10.8) | 1.04 (0.91-1.18) |
| No LLD treatment |  |  |  |  |
| LDL-C <3.35 mmol/L | 9084 | 1363 | 11.7 (11.1-12.3) | 1.00 (ref.) |
| LDL-C ≥3.35mmol/L | 12312 | 1969 | 13.2 (12.6-13.8) | 0.98 (0.91-1.05) |
| LLD treatment |  |  |  |  |
| LDL-C <3.35mmol/L | 3793 | 502 | 9.5 (8.7-10.4) | 1.00 (ref.) |
| LDL-C ≥3.35mmol/L | 15905 | 2236 | 10.4 (10.0-10.9) | 0.98 (0.89-1.08) |
| No LLD treatment |  |  |  |  |
| ApoB <1.39 g/L | 19586 | 3038 | 12.3 (11.9-12.8) | 1.00 (ref.) |
| ApoB ≥1.39 g/L | 1810 | 294 | 14.8 (13.2-16.5) | 1.00 (0.89-1.13) |
| LLD treatment |  |  |  |  |
| ApoB <1.39 g/L | 14678 | 1918 | 9.8 (9.4-10.2) | 1.00 (ref.) |
| ApoB ≥1.39 g/L | 5020 | 757 | 11.5 (10.8-12.4) | 1.03 (0.95-1.12) |
| No LLD treatment |  |  |  |  |
| ApoB/ApoA-I ratio <0.94 | 19371 | 2981 | 12.2 (11.8-12.7) | 1.00 (ref.) |
| ApoB/ApoA-I ratio ≥0.94 | 2025 | 351 | 15.4 (13.9-17.2) | 1.06 (0.95-1.19) |
| LLD treatment |  |  |  |  |
| ApoB/ApoA-I ratio <0.94 | 14837 | 1988 | 9.7 (9.3-10.2) | 1.00 (ref.) |
| ApoB/ApoA-I ratio ≥0.94 | 4861 | 750 | 11.8 (11.0-12.7) | 1.06 (0.98-1.16) |

^*^Adjusted for age, sex, and socio-economic status.

AF = atrial fibrillation; ApoA-I = apolipoprotein A-I; ApoB = apolipoprotein B; LDL-C = low-density lipoprotein cholesterol; SD = standard deviation; TC= total cholesterol; HF=heart failure; CHD=coronary heart disease; LLD=lipid-lowering drugs
